# Valorizing Agro-Industrial By-Products for Sustainable Cultivation of *Chlorella sorokiniana*: Enhancing Biomass, Lipid Accumulation, Metabolites, and Antimicrobial Potential

**DOI:** 10.3390/metabo15030212

**Published:** 2025-03-20

**Authors:** Elia Lio, Carlo Esposito, Jacopo Paini, Stefano Gandolfi, Francesco Secundo, Gianluca Ottolina

**Affiliations:** 1 Institute of Chemical Sciences and Technologies “Giulio Natta”, National Research Council of Italy, via Mario Bianco 9, 20131 Milan, Italy; jacopo.paini@scitec.cnr.it (J.P.); stefano.gandolfi@scitec.cnr.it (S.G.); 2 Department of Pharmaceutical Sciences, University of Milan, via Mangiagalli 25, 20133 Milan, Italy; elia.lio@scitec.cnr.it (E.L.),

**Keywords:** green organic solvent, antimicrobial, bioactive compounds, microalgae, agro-industrial by-products

## Abstract

**Background/Objectives:** Mixotrophic cultivation of microalgae using agro-industrial by-products as supplements offers a sustainable strategy to enhance biomass production and bioactive compound synthesis. This study aimed to evaluate the effects of different agro-industrial by-products—orange peel extract, *Cladophora glomerata* macroalgal hydrolysate, and solid-state fungal fermentation hydrolysate—on the growth and bioactivity of *Chlorella sorokiniana*. **Methods:** Microalgae were cultivated under mixotrophic conditions with different agro-industrial by-products as organic carbon sources. Biomass accumulation was monitored through dry weight measurements. Lipid extraction was carried out using dimethyl carbonate. The antimicrobial activity of the extracted compounds was assessed against *Escherichia coli*, *Bacillus megaterium*, and *Bacillus subtilis* by determining the minimal inhibitconcentrations. **Results:** Orange peel extract supplementation resulted in the highest biomass production. It increased dry weight by 13.86-fold compared to autotrophic conditions. *Cladophora glomerata* macroalgal hydrolysate followed with a 5.79-fold increase, and solid-state fungal fermentation hydrolysate showed a 4.14-fold increase. The lipophilic fraction extracted from microalgal biomass showed high yields. Orange peel extract supplementation achieved the highest extraction yield (274.36 mg/g DW). Antimicrobial activity varied based on the supplement used: biomass cultivated with orange peel extract exhibited superior activity against *E. coli*, whereas *Cladophora glomerata* macroalgal hydrolysate biomass demonstrated potent activity against *B. subtilis* (MIC: 5.67 g/mL). **Conclusions**: These findings underscore the potential of agro-industrial by-products for enhancing microalgal biomass and metabolite production. The observed antimicrobial properties highlight the application of microalgal-derived compounds in sustainable bioprocesses, supporting their use in pharmaceutical and biotechnological applications.

## 1. Introduction

Microalgae, particularly *Chlorella sorokiniana*, have recently gained significant attention. Their multifaceted applications and potential contributions to a sustainable bioeconomy are key reasons [1]. *C. sorokiniana*, a fast-growing microalga, is a promising candidate for diverse industrial applications, including biofuels, high-value biomolecules, and wastewater treatment [2]. *C. sorokiniana* stands out among microalgae for its robust adaptability to various cultivation conditions, high growth rates, and ability to utilize a wide range of organic carbon sources, which is particularly advantageous in resource recovery and bioconversion processes [3]. Its capability to grow mixotrophically, i.e., by simultaneously utilizing light and organic carbon, offers a unique opportunity to valorize agro-industrial waste streams, transforming them into high-value products while reducing the environmental footprint of waste disposal [4].

The mixotrophic growth mode of *C. sorokiniana* is facilitated by its metabolic flexibility. This allows the assimilation of organic carbon compounds through glycolysis and the oxidative pentose phosphate pathway, in addition to photosynthesis [5]. This dual metabolic strategy not only enhances biomass productivity but also enables the efficient utilization of sugar-rich waste substrates, which are typically abundant in agro-industrial by-products. The mixotrophic approach often reduces the reliance on energy-intensive light sources, further lowering operational costs [6]. In this context, exploring the potential of specific agro-industrial wastes as substrates for microalgal growth is a critical step toward the development of sustainable and economically viable processes.

The economic and industrial implications of using these residues go beyond waste management. Integrating waste valorization into microalgal bioprocesses allows industries to cut raw material costs. It also enhances operational sustainability and opens access to lucrative markets for eco-friendly products [7]. Biomass derived from *C. sorokiniana* grown on agro-industrial waste holds immense potential to produce bioactive molecules. These compounds can be further developed into therapeutic agents, drug delivery systems, or immunomodulatory supplements, addressing critical needs in modern medicine while aligning with global sustainability goals by reducing landfill waste, lowering greenhouse gas emissions, and promoting resource efficiency [8,9,10,11].

A key step in the downstream processing of microalgal biomass is the extraction of metabolites, such as lipids and antimicrobial metabolites, which often requires the use of organic solvents. Conventional solvents like chloroform and methanol are highly effective but pose significant environmental and health risks due to their toxicity and non-biodegradability [12,13]. Moreover, these traditional extraction methods are often laborious and time-consuming, while also exhibiting low selectivity and poor yields [14]. Green solvents such as dimethyl carbonate (DMC) have emerged as sustainable alternatives, offering comparable extraction efficiency with a reduced ecological footprint [15,16]. DMC is characterized by its low toxicity, high biodegradability, and relatively low environmental impact, making it an ideal candidate for industrial applications [17]. DMC aligns with increasing regulatory pressures, such as those outlined by the European REACH directive and the U.S. Environmental Protection Agency (EPA), which encourage the adoption of greener industrial practices [18,19].

In this study, we identified three distinct types of biowaste, including agro-industrial orange peel residues, the environmental biowaste *Cladophora glomerata* macroalga hydrolysate (MA-Hyd), and the solid-state fungal fermentation waste hydrolysate (SSF-Hyd). These residues are promising candidates for the mixotrophic growth of microalgae due to their high nutrient content, global availability, and potential cost benefits, as they may even be available at negative costs, with industries willing to pay for their removal [20].

Orange peel extract (OPE) is a by-product of the citrus juice industry. OPE is rich in sugars, organic acids, and essential micronutrients that are ideal for microalgal growth [21,22]. Annually, millions of tons of citrus peels are discarded, posing significant environmental challenges due to their high moisture and carbohydrate content, which can lead to rapid microbial degradation and methane emissions in landfills [23]. The valorization of OPE not only mitigates these issues but also creates an opportunity to recover valuable resources [24,25].

*C. glomerata* macroalga hydrolysate obtained by enzymatic or chemical hydrolysis contains polysaccharides, amino acids, and minerals that support microalgal growth. Macroalgal waste from the food and cosmetic industries is often underutilized, despite its high potential for bioconversion into biofuels and bioactive compounds [26]. Integrating MA-Hyd into microalgal cultivation systems could further expand the utility of these renewable resources.

Solid-state fungal fermentation waste (SSF) is generated from the biotechnological production of enzymes, bioethanol, and other fungal-derived products. It is typically rich in partially degraded carbohydrates and organic compounds, making it an attractive substrate for microalgae. Repurposing SSF waste addresses waste management challenges while contributing to a circular bioeconomy model [27].

This study aimed to investigate the link between microalgal biotechnology and waste valorization by analyzing the mixotrophic growth of *C. sorokiniana* in media supplemented with OPE, MA-Hyd, or SSF. Furthermore, it sought to evaluate the fatty acid profile of the resulting microalgal biomass and the antimicrobial activity of its extract, with the goal of establishing a correlation between the influence of the aforementioned supplements on fatty acid composition, the extract’s chemical profile, and its antimicrobial properties.

## 2. Materials and Methods

### 2.1. Supplement Preparation and Pretereatment of Biowaste Residues

The agro-industrial by-products used as nutrient supplements included dry orange (Tarocco variety) peel waste (OPW), macroalgae *Cladophora glomerata* (MA) provided by the research group of Prof. Izabela Michalak at Wrocław University of Science and Technology, and solid-state fungal fermentation (SSF) residues, obtained after fungal growth on starchy agro-industrial residues.

Before being used as a supplement, MA and SSF biomass were subjected to a hydrothermal hydrolysis process. In both cases, 10 g of dry biomass was thoroughly washed with 3 × 50 mL distilled water to remove impurities. The washed biomass was then subjected to thermal hydrolysis in a high-pressure reactor system (BR-300) equipped with a BTC-3000 temperature controller and a polytetrafluoroethylene (PTFE) liner (Berghof, Germany) under the following conditions: (i) 170 °C for 1 h with a 2% NaOH solution (*w*/*w*) for MA residue; (ii) 170 °C for 1 h with a 2% H_2_SO_4_ solution (*w*/*w*) for SSF residue.

Orange peel waste, a by-product of juice production, was shredded, mixed with water, and agitated overnight. The mixture was filtered using Whatman No. 1 paper and centrifuged (5000 rpm, 1 h), and the liquid fraction (OPE) was concentrated with a rotary evaporator and used as a supplement without any further treatment.

### 2.2. DNS Assay

Reduced sugar concentrations were determined using the 3,5-dinitrosalicylic acid (DNS) method [28]. The DNS reagent was prepared by dissolving 0.25 g of DNS in 12.5 mL of Milli-Q water, adding 7.5 g sodium potassium tartrate tetrahydrate and 5 mL of 2 M NaOH, and diluting to a final volume of 25 mL. Appropriate sample volumes of the liquid fractions of the biomasses pretreated as described in Section 2.1 were diluted with Milli-Q water (final volume: 1 mL) and mixed with 330 µL of DNS reagent, heated at 98 °C for 5 min (300 rpm), cooled on ice, and centrifuged at 13,000 rpm for 3 min. Next, a 200 µL aliquot was diluted with Milli-Q water (final volume: 3 mL), and absorbance was measured at 540 nm. A calibration curve was prepared using glucose standard solutions obtained from serial dilutions of a 2.5 mg/mL stock solution.

### 2.3. Growth of Microalgae Strain

The growth of *C. sorokiniana* (CCAP 211/8K), purchased by the Culture Collection of Algae and Protozoa (Dunbeg, OBAN, Scotland, United Kingdom), was carried out under autotrophic conditions (without supplements) or mixotrophic conditions (with supplements prepared as described in Section 2.1). Cultures were maintained at 25 °C and 120 rpm in a thermo-shaker Eppendorf Innova 42 (Eppendorf, Hamburg, Germany)) equipped with a photosynthetic lamp. Illumination followed a circadian cycle of 14 h of light and 10 h of darkness, with an intensity of 24 µmol m^−2^ s^−1^ (emission spectrum: 360–760 nm; peaks at 450 nm and 680 nm).

For 24-well plate experiments, in each well, 1 mL of *C. sorokiniana* inoculum in BG-11 medium (OD_680_ = 0.2) was mixed with 1 mL of extract solution to give final extract concentrations of 0, 1, 3, 5, 7, and 10% (*v*/*v*) in BG-11 and a final volume of 2 mL. The extract solutions from orange peel waste (OPE), solid-state fermentation residue hydrolysate (SFF-Hyd), and macroalgae hydrolysate (MA-Hyd) were initially prepared at 20% in BG-11 medium following the protocol of Kirrolia [29], then centrifuged, filtered, and diluted to the desired concentrations before autoclaving. Cultures showing the highest OD_680_ nm (measured by a Multiskan SkyHigh Microplate Spectrophotometer (Thermo Scientific, Waltham, MA, USA)) after seven days of cultivation were selected for growth in flasks. All conditions were tested in quadruplicate to ensure reproducibility and accuracy.

When microalgal growth was carried out in flask, BG-11 medium was used for autotrophic cultures, while mixotrophic cultures included 5% (*v*/*v*) of supplements (OPE, SFF-Hyd, or MA-Hyd). The pre-inoculum was prepared by suspending a single colony from BG-11 agar plates (2% *w*/*v* agar) into 100 mL of BG-11 medium. Once it reached an OD_680_ of 0.8 (value measured by a UV-Vis spectrophotometer (Jasco V-730, Jasco, Tokyo, Japan)), the inoculum was added to 150 mL of autoclaved BG-11 medium only (autotrophic condition) or BG-11 medium supplemented with additives (mixotrophic condition) in a 500 mL flask.

Cultures were maintained in triplicate, and biomass was harvested at the stationary phase after 23 days. Every 7 days, 1 mL of microalgae culture was withdrawn for (i) pH measurement, (ii) optical density (OD_680_), and (iii) dry weight (DW) analysis. For OD_680_ measurements, the absorbance spectrum of the corresponding blank for each supplement (OPE, SFF-Hyd, and MA-Hyd) was subtracted to ensure accuracy. To determine DW, 1 mL of culture was centrifuged, the supernatant was discarded, and the biomass pellet was freeze-dried before weighing.

### 2.4. Preparation of Extracts with Green Organic Solvents

For extraction, 70 mg of microalgae biomass was suspended in a dimethyl carbonate (DMC)–water (80:20 *v*/*v*) mixture. Then, it was placed on ice and subjected to 3 cycles (2 min each) of sonication using an Omni Ruptor 250-Watt Ultrasonic Cell Disruptor operating at 50% of the maximum sonication power. Following sonication, the organic phase was collected, and the DMC was subsequently removed using a rotary evaporator. The same procedure was applied to the control samples without microalgae.

### 2.5. Antimicrobial Tests

#### 2.5.1. Agar Well Diffusion Method

The agar well diffusion method was performed by inoculating the entire surface of Mueller–Hinton agar plates with microbial cultures of *Escherichia coli*, *Bacillus subtilis*, and *Bacillus megaterium*. The inoculum density was standardized to 0.5 McFarland [30]. Aseptically, wells with a diameter of 7 ± 1 mm were created in the agar using a sterile cork borer or tip. Next, 50 µL of a 1 mg/mL solution of the different extracts (obtained as described in Section 2.3) in 300 µL of a 5% Tween-20 and 10% DMSO aqueous solution was dispensed into the wells, and the agar plates were then incubated at 25 °C for 24 h. A 1 mg/mL solution of vancomycin was used as a positive control. The extract components or vancomycin diffusion into the agar medium formed a round halo where microbial growth was inhibited.

#### 2.5.2. Broth Dilution Method

The broth dilution method entailed preparing 96-well microtiter plate wells containing 0.15 mL of extract in a water solution containing 5% of DMSO and 10% of Tween-20, with a final extract concentration of 0 (control), 12.5, 25, 62.5, 125, 250, or 500 µg/mL. Next, 0.15 mL of microbial inoculum was added to each well (*E. coli*, *B. megaterium*, and *B. subtilis* at 0.5 McFarland). Subsequently, the plates were incubated for 24 h (25 °C, 100 rpm). Optical density was measured at 600 nm, and the minimum inhibitory concentration (MIC) was determined using a dose–response curve (DRC) model implemented in R [31,32].

### 2.6. Characterization of Extracts Using GC-MS

The extracts and their controls were analyzed by gas chromatography coupled to mass spectrometry (GC-MS, ThermoQuest Trace chromatograph GC—Finnigan Trace DSQ MS, Thermo Fisher Scientific, Waltham, MA, USA)equipped with a MEGA-1 MS 30 m × 0.25 mm × 0.25 µm Crossbond column, (MEGA, Legnano, Italy)). The chromatographic analysis was conducted by heating from 60 °C (initial time: 0.30 min) to 280 °C (heating rate: 8 °C/min; final time: 27.5 min) and 15 °C/min to 310 °C, with a split ratio of 50 and a column flow of 1.2 mL/min.

### 2.7. Analysis of the Lipid Profiles of the Extracts

Lipid extracts, resuspended in 1 mL of chloroform, were subjected to methylation using 100 μL of each sample and 1 mL of methanol containing NaOH (0.035 M) and incubated at 30 °C for 18 h. Subsequently, 1 mL of 15% (*v*/*v*) H_2_SO_4_ in methanol was added, and the mixture was agitated at 30 °C for 4 h [33]. The lipophilic fraction was extracted using petroleum ether and analyzed by GC-FID (Agilent Technologies 6850, Santa Clara, CA, USA) equipped with a MEGA 5 MS capillary column (30 m × 0.25 mm; film thickness: 0.25 μm). Analyses were performed in split mode (1:50) with a constant column flow of 1.2 mL/min and the following temperature program: 75 °C for 0.5 min, a ramp of 10 °C/min to 200 °C (7 min), followed by 10 °C/min to 310 °C (1 min), and a final hold at 315 °C for 1 min. Transesterified samples (2 μL, resuspended in petroleum ether, Fluka, Buchs, Switzerland) were compared against methyl ester standards (Supelco, Bellefonte, PA, USA), , including C14:0, C16:0, C18:0, C18:1, C18:2, C18:3, and C20:5.

### 2.8. Statistical Analysis

All analyses were conducted using R statistical software (v4.2.1; R Core Team 2022) [34]. Initially, one-way and two-way analysis of variance (ANOVA) were applied [35], followed by multiple-comparison tests (Tukey and Duncan tests) to discern significant differences among the extracts [35,36].

## 3. Results and Discussion

### 3.1. Pretreatment Efficacy and Sugar Releasee

Hydrothermal hydrolysis of SSF residues at 170 °C with 2% H_2_SO_4_ resulted in the highest sugar release (99.15 ± 4.05 mg/mL), highlighting its efficiency in substrate breakdown. Similarly, alkaline hydrothermal hydrolysis (170 °C, 2% NaOH) applied to MA substrates released 7.8 ± 1.95 mg/mL of reducing sugars, effectively disrupting the *C. glomerata* matrix. In the case of OPE supplementation, an average sugar concentration of 20.25 ± 2.1 mg/mL was obtained, demonstrating its potential as a nutrient-rich supplement that does not require additional chemical treatments.

### 3.2. Microalgae Growth Performance

#### 3.2.1. Twenty-Four-Well Plate Experiments

**Figure 1:**
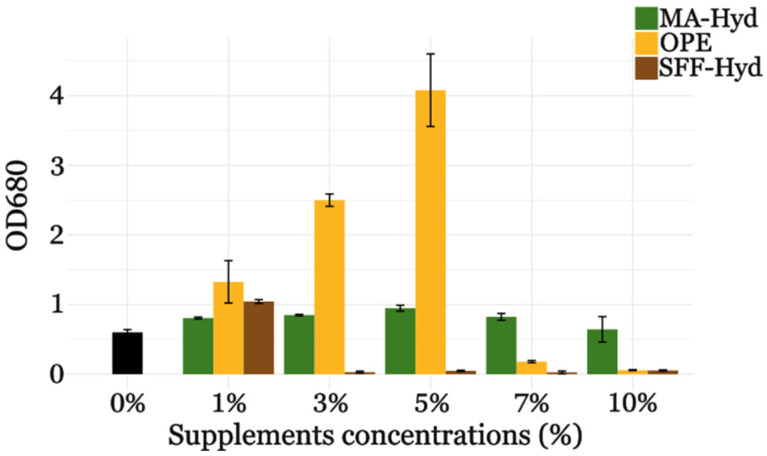
Bar graph representing the optical density at 680 nm (OD_680_) of *C. sorokiniana* culture media supplemented with different concentrations of orange peel extract (OPE), macroalgae hydrolysate (MA-Hyd), or hydrolyzed solid-state fermentation waste of fungi (SSF-Hyd) after 7 days of incubation in 24-well plates. The growth in autotrophic conditions (black) is without supplement (0%).

The growth of *C. sorokiniana* in 24-well plates, as shown in Figure 1, demonstrated a clear concentration-dependent response to the three substrates (SSF-Hyd, MA-Hyd, and OPE). The highest growth rates were observed with 5% extract concentration for both MA-Hyd and OPE, while 1% was optimal for SSF-Hyd. At concentrations exceeding 10%, growth was significantly inhibited, likely due to substrate-associated toxicity or osmotic stress, as evidenced by reduced optical density measurements.

In support of these observations, a two-way ANOVA (Table S1) was conducted to evaluate the effects of supplement type and concentration on the growth of *C. sorokiniana*. The analysis revealed significant differences for the supplement type (*p* = 0.0002), the concentration (*p* = 0.0002), and their interaction (*p* = 0.0002). These statistical results confirm that both the type of supplement and its concentration play an important role in determining the growth patterns observed.

Based on these findings, 5% supplement was selected as the standard concentration for further experiments, as it provided the best balance between promoting growth and avoiding inhibitory effects. This concentration was subsequently used to evaluate the impact of the different supplements on growth, lipid profile, and potential variations in antimicrobial activity.

#### 3.2.2. Flask Cultures

**Figure 2:**
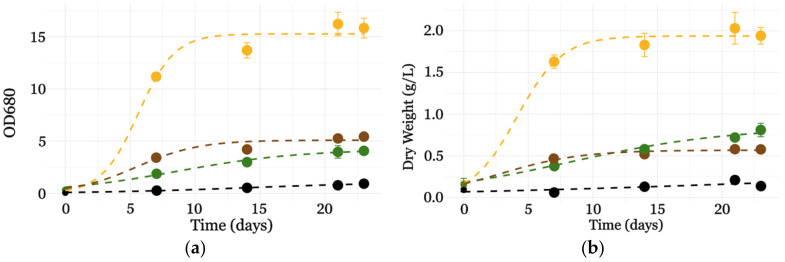
Growth curves of *C. sorokiniana* in autotrophic conditions (black circles) and in the presence of 5% orange peel extract (yellow circles), hydrolyzed macroalgae (green circles), and hydrolyzed solid-state fermentation waste of fungi (brown circles), expressed as (**a**) OD680 or (**b**) dry weight (mg/mL DW). Values are means ± SDs, n = 3. Dashed lines represent the growth kinetics that were modelled using a logistic function.

In flask cultures, the growth of *C. sorokiniana* after 23 days showed the highest OD_680_ in the case of OPE-supplemented cultures (16.22 ± 1.13), compared to MA-Hyd (4.07 ± 0.16) and SSF-Hyd (5.43 ± 0.07), while autotrophic conditions (AUTO) exhibited the lowest OD_680_ (0.92 ± 0.17) (Figure 2). Analogously, it was observed that OPE, SSF-Hyd, and MA-Hyd induced a significant increase in biomass compared to the autotrophic condition (AUTO). OPE supplementation resulted in a remarkable 13.9-fold increase, followed by 5.8-fold and 4.1-fold increases for MA and SSF-Hyd, respectively (Table 1).

OPE’s superior performance likely stems from its well-balanced nutrient profile. It provides essential macro- and micronutrients that support mixotrophic growth. This composition may enhance metabolic flexibility, promoting biomass accumulation more efficiently than other tested supplements. In contrast, the limited increase observed with SSF-Hyd and MA-Hyd may be linked to differences in nutrient availability or sugar composition. On the other hand, thanks to the capacity of *C. sorokiniana* to grow autotrophically, complete sugar consumption would not limit biomass increase. Therefore, any observed growth limitations must be attributed to other factors, including insufficient phosphate or nitrogen availability. The cultures reached the stationary phase by day 14, indicating that nutrient availability and substrate properties critically influenced biomass accumulation. These results emphasize the significant role of OPE in promoting microalgal growth under mixotrophic conditions. A two-way ANOVA (Table S1) was conducted to evaluate the effects of supplement type and cultivation time on the growth of *C. sorokiniana*, measured both as optical density (OD680) and dry weight (DW). The analysis revealed highly significant effects of supplements (*p* < 0.001) and days (*p* < 0.001), as well as their interaction (*p* < 0.001) for both growth parameters. The significant interaction terms (*p* < 0.001) underscore the dynamic influence of supplements over time, with OPE consistently driving the highest growth across both metrics. These results emphasize the critical role of supplement composition and temporal factors in optimizing *C. sorokiniana* biomass production. Although specific studies on the long-term stability of *C. sorokiniana* are limited, previous research indicates that cultivation duration, as well as factors such as inoculum size and light intensity, can influence lipid production and overall metabolic profiles. Lu et al. reported that different inoculum concentrations over a 20-day period significantly affected lipid productivity, suggesting that extended cultivation can modulate the metabolic behavior of microalgal cells [37]. Similarly, Li et al. (2015) showed that prolonged nitrogen deprivation enhances lipid accumulation, partly due to starch degradation and the reallocation of other cellular components. These findings suggest that *C. sorokiniana* may undergo metabolic adaptations in extended growth conditions, potentially affecting metabolite stability and cell viability. Further investigations are needed to better understand these dynamics and optimize cultivation strategies for biotechnological applications [38].

#### 3.2.3. pH Variation

**Figure 3:**
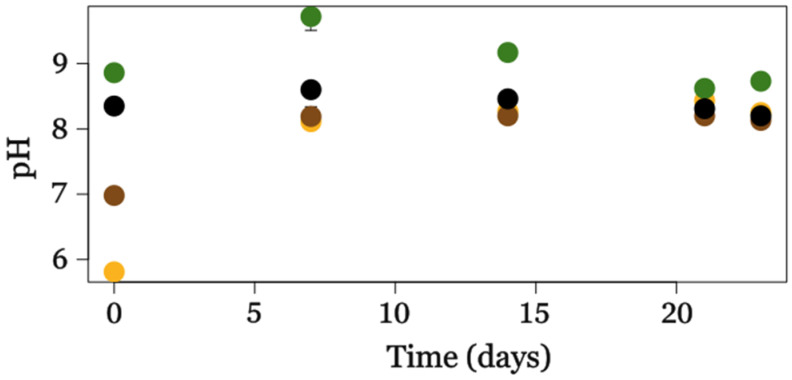
pH variation of *C. sorokiniana* cultures supplemented with different substrates (colors refer to the same samples like in Figure 2) over a 23-day incubation period.

The pH dynamics during cultivation revealed distinct trends depending on the substrate supplementation (Figure 3). The pH of the cultivation media was adjusted to 7.2 before autoclaving, and after autoclaving, the pH values varied, possibly due to heat-induced changes in the media. Despite these initial discrepancies, the microalgae were able to regulate the pH through their metabolic processes, indicating their resilience in maintaining favorable growth conditions. Notably, after 23 days of cultivation, the pH values in all tested conditions converged to 8.4, suggesting a stabilization of the system over time.

The pH value plays a crucial role in the metabolism of *C. sorokiniana*, directly influencing the regulation of specific metabolic pathways.

In the autotrophic condition, pH remained relatively stable throughout the experiment, ranging from 8.35 ± 0.10 to 8.20 ± 0.02, reflecting the absence of exogenous carbon sources and lower metabolic activity. It is worth emphasizing that, under autotrophic conditions, photosynthesis is the primary driver of growth, and the activity of key carbon fixation enzymes, such as RuBisCO, is highly sensitive to pH variations, with optimal performance around neutral to slightly alkaline values [39].

Cultures supplemented with OPE exhibited an initial acidic pH (5.81 ± 0.05), which increased steadily to a peak of 8.43 ± 0.09 on day 21 before stabilizing at 8.25 ± 0.03 by day 23. This pronounced increase could be attributed to the utilization of organic acids and sugars in the OPE, leading to the release of alkaline by-products during metabolic activity [40].

Similarly, SSF-Hyd-supplemented cultures showed a moderate increase in pH, starting at 6.98 ± 0.02 and stabilizing near neutrality (8.13 ± 0.10), potentially due to the slower assimilation of substrates and reduced metabolic by-product release.

MA-Hyd cultures began with the highest initial pH (8.86 ± 0.01), peaking at 9.72 ± 0.21 on day 7, followed by a gradual decline to 8.73 ± 0.08 by day 23, which might be linked to the buffering capacity of MA-Hyd-derived compounds or accumulation of metabolic acids.

Statistical analysis (two-way ANOVA, reported in Table S1) confirmed significant effects of both supplements (*p* < 0.001) and cultivation time (*p* < 0.001) on pH variation.

In mixotrophic conditions, the availability of organic carbon sources alters the metabolic balance, affecting the interplay between mitochondrial respiration and photosynthesis [41]. Recent studies have demonstrated that mixotrophic metabolism in *C. sorokiniana* enhances metabolic flexibility, with a coordinated activation of both photosynthetic and catabolic pathways to maximize biomass accumulation and metabolite production [5]. Additionally, pH modulates the assimilation of essential nutrients such as nitrogen and phosphorus, which play a key role in regulating lipid and starch accumulation [42,43]. The stabilization of pH observed in our cultures after 23 days suggests that *C. sorokiniana* possesses compensatory metabolic mechanisms in response to initial fluctuations, allowing optimal adaptation to the environmental conditions provided by the different supplements.

### 3.3. Green Extraction Yields (Lipophilic Compounds)

The extraction of lipophilic fractions from microalgae has traditionally relied on organic solvents such as chloroform, methanol, and hexane, which, despite their effectiveness, pose significant environmental and safety concerns due to their toxicity, volatility, and persistence in the environment [44]. While alternative green solvents have been explored, their extraction efficiency and industrial applicability remain key challenges. Among them, dimethyl carbonate (DMC) has emerged as a promising candidate due to its low toxicity, high biodegradability, and favorable physicochemical properties for lipid extraction [45].

DMC was selected over other green solvents, such as ethanol and ethyl acetate, based on its polarity, compatibility with microalgal lipids, and ability to selectively extract metabolites with minimal co-extraction of unwanted contaminants. Moreover, its higher vapor pressure compared to conventional solvents facilitates solvent recovery, improving process sustainability and reducing operational costs.

**Figure 4:**
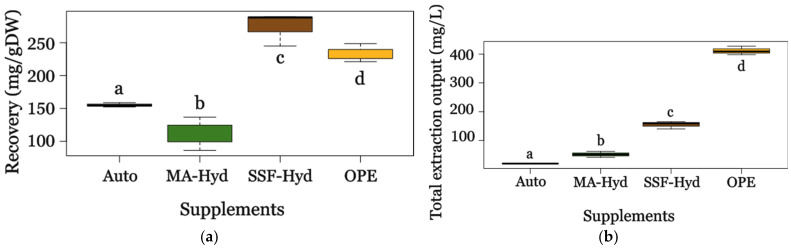
Extraction yields (**a**) and total extraction output (**b**) of the lipophilic fraction from *C. sorokiniana* biomass cultivated in different conditions (for extract abbreviations, see Figure 1. Autotrophic conditions-Auto). Small lettering according to Duncan’s test.

The extraction yields obtained using dimethyl carbonate (DMC) varied significantly among the different cultures (Figure 4a). The highest yield was observed in SFF-Hyd-supplemented cultures (274.36 ± 25.49 mg/g DW), followed by OPE (233.50 ± 13.94 mg/g DW), while autotrophic cultures and MA-Hyd supplementation exhibited the lowest yields, with 155.29 ± 3.26 mg/g DW and 111.76 ± 25.33 mg/g DW, respectively.

The higher effective extraction in SFF-Hyd- and OPE-supplemented cultures can be attributed to their richer composition of lipophilic compounds, including intracellular secondary metabolites and lipids, as supported by the subsequent analysis of fatty acid profiles. By contrast, the lower yields observed under autotrophic and MA-Hyd conditions could result from a reduced accumulation of extractable compounds, possibly due to nutrient limitations or differences in metabolic pathways. The variability in yields highlights the impact of substrate composition and cultivation conditions on the biochemical profile of the biomass and its suitability for extraction. These findings align with those of the authors of [46], who noted that the availability of organic substrates plays a crucial role in determining the biochemical composition of microalgal biomass, influencing the efficiency of subsequent extraction processes.

A one-way ANOVA (Table S1) confirmed a significant effect of the culture conditions on extraction yields (*p* < 0.001), with post hoc analysis revealing that SFF-Hyd-supplemented cultures yielded significantly more than OPE, autotrophic, and MA-Hyd conditions. This result is consistent with previous studies [47,48] that demonstrated the enhanced accumulation of lipophilic compounds in biomass supplemented with organic substrates. The higher yields in the SFF-Hyd and OPE cultures suggest that these substrates enhance the production of lipophilic compounds, likely due to their composition promoting the accumulation of lipids and secondary metabolites, as observed by Abreu [49]. However, when considering the total extraction output per fermentation (Figure 4b), a different perspective emerges, highlighting the industrial relevance of the process beyond specific yield efficiency. OPE-supplemented cultures showed the highest total extract recovery (411.62 ± 14.94 mg/L), followed by SFF-Hyd (154.94 ± 13.15 mg/L), MA-Hyd (51.56 ± 10.34 mg/L), and autotrophic conditions, which yielded only 19.66 ± 0.63 mg/L. These findings highlight the importance of integrating both extraction efficiency (mg/gDW) and total process yield (mg/L) when assessing the scalability of bio-based production. While SFF-Hyd exhibited the highest efficiency in terms of mg/gDW, OPE supplementation proved to be the most promising condition for large-scale production due to the significantly higher total yield of extracted lipophilic compounds.

These results highlight OPE as a valuable substrate not only for enhancing lipid accumulation but also for maximizing the absolute recovery of metabolites, making it an attractive candidate for industrial applications. The adoption of DMC in combination with the valorization of agro-industrial waste underscores its potential for a more sustainable bio-based economy. Not only does its use enhance the eco-friendliness of extraction processes, but it also aligns with stringent safety and environmental standards, improving both scalability and industrial feasibility. Additionally, the economic advantages of solvent recovery and recycling further contribute to the cost-effectiveness of this approach [50].

### 3.4. Antimicrobial Activity Assessed by the Agar Well Diffusion Method

**Figure 5:**
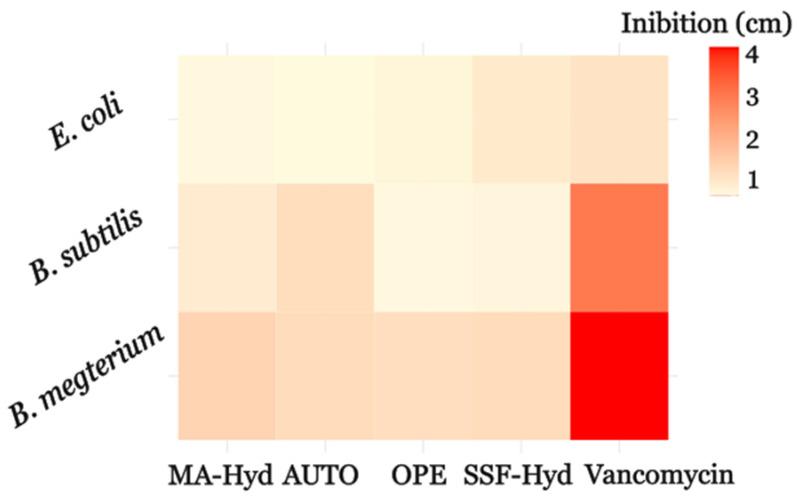
Heatmap of inhibition zones showing the antimicrobial activity of *C. sorokiniana* extracts. Values are diameters of the inhibition zones against *E. coli*, *B. megaterium*, and *B. subtilis*. For extract abbreviations, see Figure 1. Autotrophic conditions (AUTO).

The antimicrobial activity of the extracts, assessed via the agar well diffusion method as a preliminary screening, revealed variability in inhibition zones among the tested bacterial strains (Figure 5). Against *E. coli*, the largest inhibition zone was observed for SFF-Hyd-supplemented cultures (1.00 ± 0.46 cm), while *B. megaterium* showed the highest sensitivity to MA-Hyd-supplemented extracts (1.40 ± 0.17 cm). For *B. subtilis*, MA-Hyd extracts also demonstrated the greatest activity (0.97 ± 0.15 cm), whereas other extracts exhibited more limited inhibition. The positive control, vancomycin, showed inhibition zones of 1.08 ± 0.30 cm, 2.60 ± 0.09 cm, and 3.48 ± 0.08 cm against *E. coli*, *B. subtilis*, and *B. megaterium*, respectively.

These differences reflect the distinct chemical compositions of the extracts, with variations in bioactive compound profiles influencing activity against Gram-positive and Gram-negative strains. A more detailed antimicrobial activity quantification of the extracts was carried out by assessing the minimum inhibitory concentrations (MICs).

### 3.5. Minimum Inhibitory Concentrations

**Figure 6:**
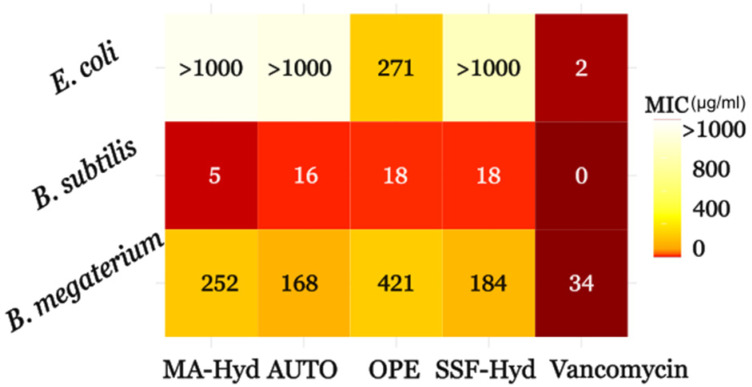
Heatmap of MIC values showing the antimicrobial activity of *C. sorokiniana* extracts. Darker shades indicate lower MICs (higher potency) against *E. coli*, *B. megaterium*, and *B. subtilis*. For extract abbreviations, seeFigure 1. Autotrophic conditions (AUTO).

The MIC values confirmed the findings from the agar well diffusion method, highlighting significant differences in the antimicrobial potency of the extracts (Figure 6). OPE extracts demonstrated the strongest activity against *E. coli* (271.43 ± 33.44 µg/mL), far superior to the other extracts (e.g., MA-Hyd: 1659.93 ± 202.29 µg/mL; autotrophic: 1517.04 ± 582.26 µg/mL). The higher activity of OPE against *E. coli* could suggest that the composition of the OPE-supplemented medium may influence the production of bioactive metabolites targeting Gram-negative bacteria. This is consistent with findings in previous studies showing that microalgae and their extracts, influenced by the composition of the growth medium, can produce bioactive molecules, including fatty acids, which are known to exhibit antimicrobial properties [51].

For *B. megaterium*, the lowest MIC was observed for MA-Hyd (252.15 ± 27.49 µg/mL), while the SFF-Hyd (184.26 ± 76.82 µg/mL) and autotrophic cultures (168.41 ± 28.68 µg/mL) displayed comparable results. OPE showed limited activity against *B. megaterium*, with an MIC of 421.65 ± 292.82 µg/mL. These results suggest that specific metabolites, which may be influenced by the type of growth medium, are responsible for the observed antimicrobial effects. In line with these findings, previous research has highlighted how the supplementation of growth substrates can significantly affect the production of metabolites in microalgae, with higher supplement concentrations promoting the production of metabolites with higher antimicrobial activity [52].

Against *B. subtilis*, MA-Hyd extracts exhibited the most potent activity (MIC = 5.67 ± 0.64 µg/mL), outperforming OPE (18.34 ± 5.62 µg/mL) and SFF (17.83 ± 1.97 µg/mL). The antimicrobial activity of MA-Hyd against *B. subtilis* suggests the presence of specific antibacterial agents favored by macroalgae-derived supplementation.

The observed differences in the antimicrobial activity of the extracts against *B. megaterium* and *B. subtilis*, despite both being Gram-positive bacteria, can be attributed to variations in the lipid composition of their cellular membranes. These differences influence membrane permeability and susceptibility to antimicrobial compounds. Previous studies have highlighted that membrane lipid composition can vary significantly among *Bacillus* species, affecting their response to environmental and antimicrobial stressors.

For instance, *B. subtilis* is known for its ability to modulate membrane lipid composition in response to stress, increasing the proportion of saturated fatty acids to reduce membrane fluidity and limit the influx of harmful compounds. This adaptability may make it less susceptible to the tested extracts [53]. Conversely, *B. megaterium* may have a less adaptable lipid composition, rendering it more vulnerable to the action of the same extracts. These differences in membrane composition and fluidity may explain the greater efficacy of the extracts against *B. megaterium* compared to *B. subtilis* [54].

These findings highlight the complex interactions between the culture conditions, substrate composition, and the bioactive potential of the extracts. It is worth emphasizing that, even though resistance to prolonged exposure to microalgal metabolites was not tested, the antimicrobial activity of the extracts should be seen as the result of multiple bioactive compounds acting together rather than a single antibiotic-like effect. This synergistic action could help prevent antibiotic resistance, making them valuable as natural and sustainable alternatives [55].

Further studies focusing on the specific metabolites produced under different growth conditions will help elucidate the mechanisms driving these antimicrobial effects.

### 3.6. Fatty Acid Methyl Ester (FAME) Profile Analysis

This study aimed to evaluate how different supplements influence the lipid profile of *C. sorokiniana* and its potential implications for antimicrobial activity. Fatty acids, particularly unsaturated ones, have been reported to play a role in antimicrobial mechanisms, either by disrupting bacterial membranes or interfering with metabolic processes. Therefore, characterizing the lipid composition of the extracts is essential to better understand their bioactive potential.

The analysis focused on eight key fatty acids, selected based on their structural diversity and biological significance. These included saturated fatty acids (SFAs)—myristic acid (C14:0), palmitic acid (C16:0), and stearic acid (C18:0)—and unsaturated fatty acids (UFAs)—palmitoleic acid (C16:1), linoleic acid (C18:2), gamma-linolenic acid (γ-C18:3), alpha-linolenic acid (α-C18:3), and oleic acid (C18:1).

The results revealed variation in the proportions of these SFAs and UFAs depending on the supplement used, which may have influenced the antimicrobial activity observed in previous experiments. These findings provide further insight into the bioactive potential of *C. sorokiniana* extracts and their possible applications in biotechnology.

As reported in Table 2, saturated fatty acids (SFAs) were most abundant in the autotrophic condition (28.8 ± 5.6%), followed by OPE (26.0 ± 1.5%), MA-Hyd (23.7 ± 1.8%), and SSF-Hyd (23.6 ± 1.9%). Unsaturated fatty acids (UFAs) accounted for the remaining fraction and represented the major component in all treatments.

**Figure 7:**
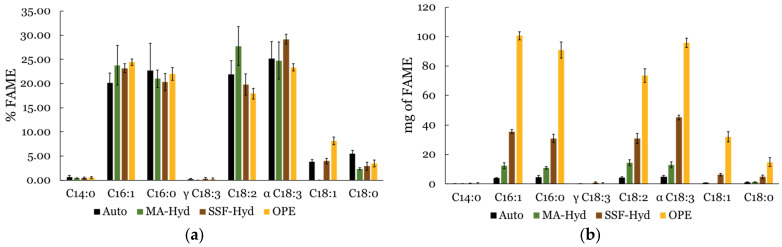
Bar plots of FAME profiles, (**a**) relative percentages and (**b**) total contents, displaying the lipid composition of *C. sorokiniana* biomass growth in autotrophic conditions (AUTO) and in the presence of 5% orange peel extract (OPE), hydrolyzed macroalgae (MA-Hyd), or hydrolyzed solid-state fermentation waste of fungi (SSF-Hyd). The abbreviations for fatty acids are as follows: C14:0 (myristic acid), C16:1 (palmitoleic acid), C16:0 (palmitic acid), γ C18:3 (γ-linolenic acid), C18:2 (linoleic acid), α C18:3 (α-linolenic acid), C18:1 (oleic acid), and C18:0 (stearic acid). Values are means ± SDs, n = 3.

The relative abundance of each fatty acid was calculated as a percentage of the total FAME content in each extract. Among the individual unsaturated fatty acids (Figure 7a), alpha-linolenic acid (α-C18:3) was the most abundant in SSF-Hyd-supplemented extracts (29.16 ± 1.05%), followed by MA-Hyd (24.74 ± 3.85%) and OPE (23.34 ± 0.77%). This compound, known for its anti-inflammatory and skin-regenerating properties, adds significant value to SSF-Hyd-derived biomass. Linoleic acid (C18:2), another key UFA with notable applications in skincare and pharmaceutical formulations, was most abundant in MA-Hyd-supplemented extracts (27.73 ± 4.00%).

For saturated fatty acids, palmitic acid (C16:0) consistently accounted for a significant proportion of total FAMEs, with the highest levels observed in autotrophic conditions (22.71 ± 5.63%). This highlights the potential of autotrophic cultivation for producing SFA-rich biomass, which is valuable in the cosmetic and surfactant industries.

While relative abundances provide insights into lipid composition, total FAME production (mg per fermentation) offers a more comprehensive view of lipid productivity (Figure 7b). OPE supplementation yielded the highest absolute amounts of all major fatty acids, surpassing SSF-Hyd and MA-Hyd, despite their higher UFA proportions. Specifically, α-C18:3 reached its highest absolute yield in OPE (95.85 ± 3.17 mg per fermentation), over twice the amount produced in SSF-Hyd (45.15 ± 1.63 mg per fermentation). Similarly, C18:2 production peaked in OPE (73.67 ± 4.61 mg per fermentation), despite its highest relative percentage being in MA-Hyd. For SFAs, C16:0 had the highest absolute production in OPE (90.82 ± 5.43 mg per fermentation), confirming OPE as the most productive condition for lipid accumulation.

The observed differences in fatty acid composition and production suggest that metabolic pathway regulation varies with supplementation. Lipid biosynthesis pathways appear to be differentially regulated based on substrate type. While SSF-Hyd and MA-Hyd appear to enhance UFA accumulation, OPE supplementation leads to a higher total lipid output, potentially reflecting differences in carbon allocation and fatty acid metabolism.

### 3.7. Lipid Profile, GC-MS Analysis, and Antimicrobial Activity Correlation Analyzed via PCA

Principal Component Analysis (PCA) was employed to investigate the relationships between the chemical composition of the *C. sorokiniana* extracts and their antimicrobial activity. This statistical technique reduces data complexity by highlighting the main sources of variability among the samples and identifying correlations between metabolites and bioactivity. In this study, PCA enabled the identification of specific molecules potentially associated with antimicrobial activity and provided insights into the role of microalgal metabolism in metabolite production. A crucial aspect of PCA interpretation is the choice of negative correlation with MIC values as an indicator of potential antimicrobial activity. Since MIC represents the minimum inhibitory concentration, lower MIC values indicate higher antimicrobial potency. Consequently, metabolites that show a negative correlation with MIC are more likely to be responsible for an antibacterial effect, as their abundance is higher in extracts with greater antimicrobial activity. Additionally, the first two principal components (PC1 and PC2) explain 83% of the total variance, providing a robust representation of the dataset.

**Figure 8:**
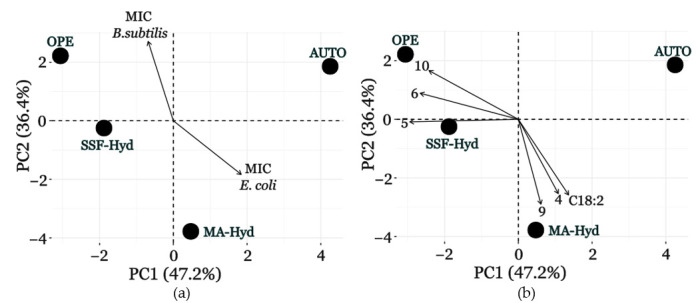
Simplified biplots of Principal Component Analysis (PCA) of MIC values, GC-MS compounds, and FAMEs for *C. sorokiniana* under different supplementation conditions. (**a**) Biplot showing MIC values and supplementation conditions. (**b**) Biplot displaying the correlation between identified metabolites and supplementation conditions. The analyzed compounds include naphthalene, 1,2,3,4-tetrahydro-2-phenyl-(4), neophytadiene (5), 3,7,11,15-tetramethyl-2-hexadecen-1-ol (6), n-hexadecanoic acid (9), and 9,12-octadecadienoic acid (10).

As shown in Figure 8a, PCA revealed a clear separation of samples based on their chemical composition. The extract derived from autotrophic cultivation (AUTO) clustered separately in the factorial plane, indicating a distinct metabolic profile compared to those obtained from heterotrophic cultures. Extracts obtained from SSF-Hyd- and OPE-supplemented cultures exhibited greater similarity, suggesting the presence of shared metabolites, whereas MA-Hyd displayed a more unique metabolic composition. These differences reflect the influence of cultivation on microalgal metabolism and the consequent production of metabolites. Through PCA, it was possible to emphasize the relationships between the chemical compositions of the extracts and the MIC values, confirming previous observations. OPE showed a negative correlation with the MIC of *E. coli*, while MA-Hyd negatively correlated with the MIC of B. subtilis. This means that extracts derived from microalgae cultivated with OPE have higher antimicrobial activity against *E. coli*, whereas those obtained with MA-Hyd are more effective against *B. subtilis*.

The molecules analyzed in the PCA were identified through GC-MS analysis with a similarity score above 80% against the NIST library and through FAME analysis (reported in Table S2). In Figure 8b, a correlation was explored between the analytical profiles of extracts obtained from different supplements and the molecules with the highest weight in the PCA. Specifically, among the compounds that showed a positive correlation with OPE, neophytadiene (5), 3,7,11,15-tetramethyl-2-hexadecen-1-ol (6), and 9,12-octadecadienoic acid (Z,Z)-(10) were highlighted. Conversely, n-hexadecanoic acid (9), 1,2,3,4-tetrahydro-2-phenyl-naphthalene (4), and C18:2 showed a positive correlation with MA-Hyd.

For a comprehensive view of the PCA analysis, refer to Figure S1, where the complete PCA plot is provided, highlighting the relationships between supplements, MIC values, and metabolites.

By combining the results of the negative correlation with MIC values and the positive correlation between supplements and metabolites, it is possible to hypothesize an antimicrobial role for the identified molecules. Neophytadiene (5) is a diterpene known for its antimicrobial properties, which potentially acts by destabilizing bacterial membranes and inhibiting microbial growth [56]. 3,7,11,15-Tetramethyl-2-hexadecen-1-ol (6) is a terpenoid alcohol that may exert antimicrobial activity by altering bacterial membrane fluidity [57]. Although its exact mechanism is not fully characterized, its structural properties suggest interactions with bacterial membrane lipids. 9,12-Octadecadienoic acid (Z,Z)-(10) is a polyunsaturated fatty acid well documented for its ability to interact with Gram-negative bacterial membranes, increasing their permeability and inducing structural damage [58]. N-hexadecanoic acid (9) is a saturated fatty acid that may exert antimicrobial effects by modifying bacterial membrane permeability and altering lipid metabolism in *B. subtilis* [59]. Naphthalene, 1,2,3,4-tetrahydro-2-phenyl-(4) is an aromatic compound that could interfere with key metabolic processes in Gram-positive bacteria, although its antimicrobial activity remains poorly characterized. Finally, C18:2 is known for its antimicrobial activity involving membrane interactions and lipid fluidity modulation [58].

PCA allowed the identification of specific molecules potentially responsible for the observed antimicrobial activity in *C. sorokiniana* extracts, highlighting the importance of integrating multivariate statistical approaches into the analysis of microalgal bioactivity. These findings demonstrate the potential of *C. sorokiniana* extracts as a source of natural antimicrobial compounds and provide a basis for future studies aimed at isolating and characterizing bioactive metabolites. Moreover, optimizing cultivation conditions could represent an effective strategy to enhance the production of these bioactive molecules, with potential applications in biotechnology and pharmaceuticals.

## 4. Conclusions

This study provides a proof of concept for leveraging microalgal biotechnology within a sustainable and application-driven framework in the context of the circular bioeconomy.

Supplement selection plays a crucial role in shaping the metabolic profile of *C. sorokiniana*, influencing biomass yield, lipid accumulation, and bioactive compound production. Integrating agro-industrial by-products into microalgal cultivation enhances productivity while aligning with sustainable bioprocessing. The feasibility of using the eco-friendly solvent dimethyl carbonate (DMC) for metabolite extraction further reinforces the potential for greener biotechnological applications.

The observed differences in lipid yield and composition underscore how external nutrient availability can direct microalgal metabolism toward specific biochemical outputs. While OPE supplementation maximized total lipid production, SSF-Hyd and MA-Hyd favored higher proportions of unsaturated fatty acids (UFAs), highlighting the potential to tailor microalgal lipid profiles particularly relevant for nutraceutical and cosmetic applications. Antimicrobial activity also emerged as an additional functional trait of the microalgal extracts, with significant variability depending on the cultivation conditions. The PCA analysis allowed us to correlate specific metabolites with antimicrobial properties, identifying compounds such as neophytadiene, n-hexadecanoic acid, and 9,12-octadecadienoic acid as possible contributors to bacterial inhibition. Notably, while some metabolites showed strong correlations with *E. coli* and *B. subtilis* inhibition, no clear association was found for *B. megaterium*, suggesting that antimicrobial effects may result from synergistic interactions rather than single compounds.

By linking substrate-driven metabolic modulation to functional bioactivities, our findings lay the foundation for developing bio-based antimicrobial agents and tailored lipid products.

## Figures and Tables

**Table 1:** *Chlorella sorokiniana* biomass production in autotrophic conditions or in the presence of supplement.

Supplement	Biomass ^1^ mg/mL (DW)	Relative Biomass Growth
AUTO	0.14 ± 0.04	1
OPE	1.94 ± 0.10	13.86
SSF-Hyd	0.58 ± 0.01	4.14
MA-Hyd	0.81 ± 0.08	5.79

^1^ After 23 days of cultivation. For culture media compositions, see section 2.3.s.

**Table 2:** Percentages of saturated and saturated fatty acids in *Chlorella sorokiniana* biomass cultivated with different supplements.

Supplements	Saturated FAs ^1^ (%)	Unsaturated FAs ^1^ (%)
AUTO	28.8 ± 5.6	71.2 ± 4.9
MA-Hyd	23.7 ± 1.8	76.3 ± 6.8
SSF-Hyd	23.6 ± 1.9	76.4 ± 2.7
OPE	26.0 ± 1.5	74.0 ± 1.7

^1^ Percentages of FAs were determined by GC-FID after derivatization to fatty acid methyl esters, as described in Section 2.7.

## Data Availability

Data are contained within the article or the Supplementary Materials.

## Supplementary Materials

The following supporting information can be downloaded at https://www.mdpi.com/article/10.3390/metabo15030212/s1: Figure S1: Complete biplot of Principal Component Analysis (PCA) illustrating the relationships between MIC values, GC-MS compounds, and FAME profiles for *C. sorokiniana* under different supplementation conditions. Correlations are highlighted in different colors: red indicates a negative correlation between metabolites and MIC for *E. coli*, while green represents metabolites negatively correlated with MIC for *B. subtilis*. For the identification of numbered molecules, refer to Table S2; Table S1: A comprehensive overview of the ANOVA results, offering valuable insights into the significance of the observed differences among the experimental groups; Table S2: List of molecules identified through GC-MS analysis and used for the principal component analysis (PCA) illustrated in Figure 8. The molecules were selected based on a “match factor” greater than 80.
