# Supplementary material for: Valorizing Agro-Industrial By-Products for Sustainable Cultivation of Chlorella sorokiniana: Enhancing Biomass, Lipid Accumulation, Metabolites, and Antimicrobial Potential
Source: Metabolites. 2025 Mar 20;15(3):212. doi: 10.3390/metabo15030212 (PMC11943808; doi:10.3390/metabo15030212)
Supplement: Supplementary file 1 [file metabolites-15-00212-s001.zip › metabolites-3517782-supplementary.pdf]

Supplementary Materials:

Valorizing agro-industrial by-products for sustainable cultivation of *Chlorella sorokiniana*: enhancing biomass, lipid accumulation, metabolites and antimicrobial potential

Elia Lio[1,2], Carlo Esposito[2], Jacopo Paini[1], Stefano Gandolfi[1], Francesco Secundo[1,*] and Gianluca Ottolina[1,*]

Index

Table S1. Provides a comprehensive overview of the ANOVA results, offering valuable insights into the significance of the observed differences among the experimental groups. Signif. codes: 0 "***", 0.001 "**", 0.01 "*", 0.05 ".", 0.1 "", 1.

Table S2. List of molecules identified through GC-MS analysis and used for the principal component analysis (PCA) illustrated in Figure 8. The molecules were selected based on a "match factor" greater than 80

Figure S1. Complete biplot of Principal Component Analysis (PCA) illustrating the relationships between MIC values, GC-MS compounds, and FAME profiles for *C. sorokiniana* under different supplementation conditions. Correlations are highlighted in different colors: red indicates a negative correlation between metabolites and MIC for *E. coli*, while green represents metabolites negatively correlated with MIC for *B. subtilis*. For the identification of numbered molecules, refer to Table S2.

Table S1. Provides a comprehensive overview of the ANOVA results, offering valuable insights into the significance of the observed differences among the experimental groups. Signif. codes: 0 "***", 0.001 "**", 0.01 "*", 0.05 ".", 0.1 "", 1.

Analysis type	Way	Groups	Df	Sum Sq	Mean Sq	F value	Pr(>F)
**24 well**	Two	Supplements	5	5.039	1.0078	111.21	0.000216 ***
		Concentrations	2	2.658	1.3292	146.66	0.000216 ***
		Supplement: Concentrations	10	3.349	0.3349	36.96	0.000216 ***
		Residuals	54	0.489	0.0091		
**OD growth**	Two	Supplements	3	727.7	242.57	51.90	7.47e-14 ***
		Days	1	227.0	226.95	48.56	2.08e-08 ***
		Supplements: Days	3	184.2	61.42	13.14	4.09e-06 ***
		Residuals	40	187.0	4.67		
**DW growth**	Two	Supplements	3	16.140	5.380	128.68	< 2e-16 ***
		Days	1	3.189	3.189	76.27	9.0e-12 ***
		Supplements: Days	3	2.782	0.927	22.18	2.2e-09***
		Residuals	52	2.174	0.042		
**pH growth**	Two	Supplements	3	14.038	4.679	26.67	1.39e-10 ***
		Days	1	2.911	2.911	16.59	0.000159 ***
		Supplements:Days	3	8.956	2.985	17.01	7.93e-08 ***
		Residuals	52	9.124	0.175		
**MIC**	Two	Supplements	4	1529210	382303	5035253	1.44e-10 ***
		Bacteria	2	2633240	1316620	17341016	2.54e-11 ***

		Bacteria: Supplements	8	2755325	344416	4536250	1.56e-10 ***
		Residuals	3	0	0		
**AWD**	Two	Bacteria	2	8.216778	4.108389	81.086	7.97e-13 ***
		Supplements	4	23.20411	5.801028	114.494	1.02e-17 ***
		Bacteria: Supplements	8	8.736556	1.092069	21.554	1.9e-10 ***
		Residuals	30	1.52	0.050667		
**Extraction yield (mg/gDW)**	One	Residuals	3	48836	16279	43.5	2.67e-05 ***
**Extraction yield (mg/L)**	One	Residuals	3	284386	94795	753	3.8e-10 ***
		Residuals	8	1006	126		

Table S2. List of molecules identified through GC-MS analysis and used for the principal component analysis (PCA) illustrated in Figure 8 and S1. The molecules were selected based on a "match factor" greater than 80

Molecules	Reference number for Fig. 8
2,4,7,9-Tetramethyl-5-decyn-4,7-diol	1
Benzene, 1,1'-(1,2-cyclobutanediyl)bis-, trans-	3
1,2,3,4-tetrahydro-2-phenyl-naphthalene,	4
Neophytadiene	5
3,7,11,15-Tetramethyl-2-hexadecen-1-ol	6
Palmitoleic acid	8
nHexadecanoic acid	9
9,12-Octadecadienoic acid (Z,Z)-	10
Heptadecanoic acid	11

Heptacosane	12
9,12,15-Octadecatrienoic acid, (Z,Z,Z)-	13
Ethanol, 2-(9-octadecenyloxy)-, (Z)-	14
Tributyl acetylcitrate	15
Benzene, 1,1'-[2-methyl-2-(phenylthio)cyclopropylidene]bis-	16
Cyclohexane, 1,3,5-triphenyl-	17
Octadecane, 3-ethyl-5-(2-ethylbutyl)-	18
9,12-Octadecadienoic acid (Z,Z)-, 2-hydroxy-1-(hydroxymethyl)ethyl ester	19
Octadecanoic acid	20
Thiocarbamic acid, N,N-dimethyl, S-1,3-diphenyl-2-butenyl ester	21
Benzene, 1,1'-(3-methyl-1-propene-1,3-diyl)bis-	22
5,6,11,12-Tetrahydrodibenz(b,f)azocine	24

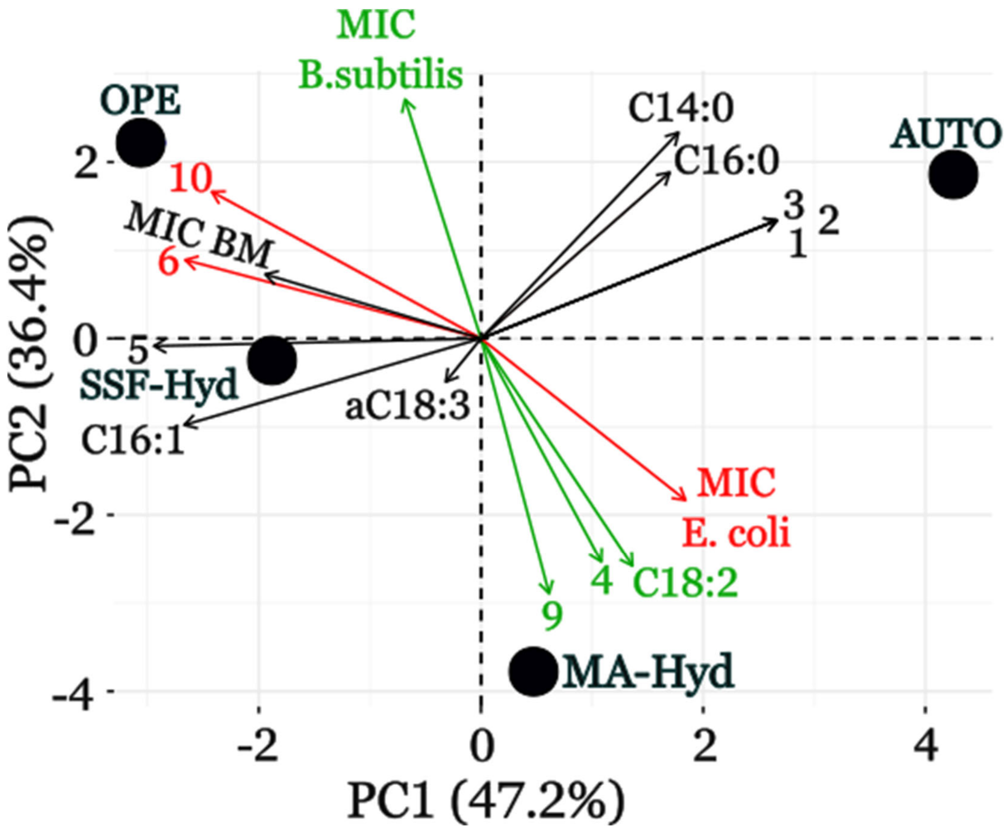

Figure S1. Complete biplot of Principal Component Analysis (PCA) illustrating the relationships between MIC values, GC-MS compounds, and FAME profiles for *C. sorokiniana* under different supplementation conditions. Correlations are highlighted in different colors: **red** indicates a negative correlation between metabolites and **MIC for *E. coli***, while **green** represents metabolites negatively correlated with **MIC for *B. subtilis***. For the identification of numbered molecules, refer to **Table S2**.
